# Heterotopic ossification in primary rectal cancer with squamous cell carcinoma-like differentiation

**DOI:** 10.20407/fmj.2021-013

**Published:** 2022-01-25

**Authors:** Yoshihiro Imaeda, Satoshi Arakawa, Hironobu Yasuoka, Hiroyuki Kato, Hidetoshi Nagata, Yukio Asano, Norihiko Kawabe, Kazuya Shiogama, Makoto Urano, Ken-ichi Inada, Tetsuya Tsukamoto, Akihiko Horiguchi

**Affiliations:** 1 Department of Gastroenterological Surgery, Fujita Health University Bantane Hospital, Nagoya, Aichi, Japan; 2 Faculty of Medical Technology, Fujita Health University, School of Health Sciences, Toyoake, Aichi, Japan; 3 Department of Pathology, Fujita Health University, School of Medicine, Toyoake, Aichi, Japan

**Keywords:** Rectal cancer, Heterotopic ossification, Squamous cell carcinoma-like differentiation

## Abstract

**Objectives:**

Heterotopic ossification (HO), which occurs when bone tissue forms outside the skeleton, is extremely rare in rectal cancer. Adenocarcinoma is the histological type of all reported primary colorectal cancers with HO. However, in the present case, we observed areas of adenocarcinoma with squamous cell carcinoma-like differentiation. Here we conducted histopathological and immunohistochemical analyses to identify the mechanisms of HO development, to differentiate between adenocarcinoma and squamous cell carcinoma-like phenotypes, and to understand the associated prognostic implications.

**Case report:**

A 62-year-old woman was admitted to our hospital with symptoms of intermittent hematochezia without abdominal pain. Colonoscopy revealed stenosis with a protuberant mass in the rectum. Abdominopelvic contrast-enhanced computed tomography showed irregular wall thickness of the rectum, multiple lymph node metastases, and liver metastases. The rectal tumor exhibited calcified deposits with marked hyperintensity. We then performed Hartmann’s operation and D3 lymph node resection. The biopsy specimen revealed tubular and solid adenocarcinoma nests and squamous carcinoma-like components over a necrotic extent without secreted mucin. She received chemotherapy (mFOLFOX6 with bevacizumab) as the first option and is alive 5 months after surgery.

**Conclusion:**

To the best of our knowledge, this is the first case of heterotopic ossification in a primary rectal cancer with squamous cell carcinoma-like differentiation that was surgically resected. This case suggests that BMP-2 transformed fibroblasts and pluripotent stem cells into osteocytes. We conclude that the squamous cell carcinoma-like lesion was squamous metaplasia of adenocarcinoma.

## Introduction

Heterotopic ossification (HO) occurs when bone tissue forms outside the skeleton. HO comprises benign and malignant neoplasms of organs such as the lung, breast, liver, pancreas, kidney, bladder, and ovary.^1–7^ HO in rectal cancer, which was first reported by Dukes^8^ in 1939, rarely occurs (suggested incidence of 0.4% in rectal cancer). Previous reports of primary colorectal cancers with HO have been diagnosed as well- or moderately differentiated adenocarcinoma.^9^ However, in the present case, we observed areas of adenocarcinoma with squamous cell carcinoma-like differentiation.

We conducted histopathological and immunohistochemical analyses to identify the mechanisms of HO development, to differentiate between the adenocarcinoma and squamous cell carcinoma-like phenotypes, and to identify associated prognostic implications. Moreover, we present a review of the relevant literature.

## Case report

A 62-year-old woman was admitted to our hospital with symptoms of intermittent hematochezia without abdominal pain. Blood tests revealed low hemoglobin (Hb) concentration (10.5 g/dL), low red blood cell count (RBC) (3.82×10^6^/μL), and mild leukocytosis. The carcinoembryonic antigen (CEA) and carbohydrate antigen (CA) 19-9 concentrations were 79.6 ng/mL and 487.0 ng/dL, respectively. Other laboratory test results were normal.

**Figure 1:**
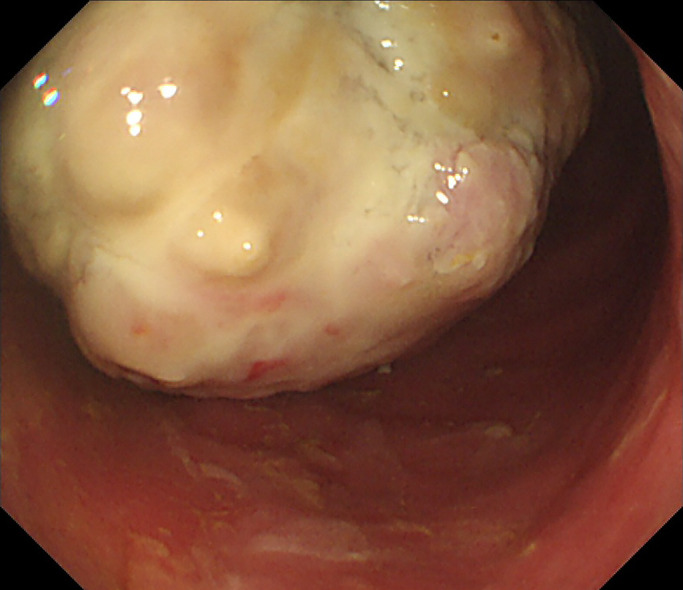
Colonoscopy reveals stenosis with a protuberant mass on the pelvis.

**Figure 2:**
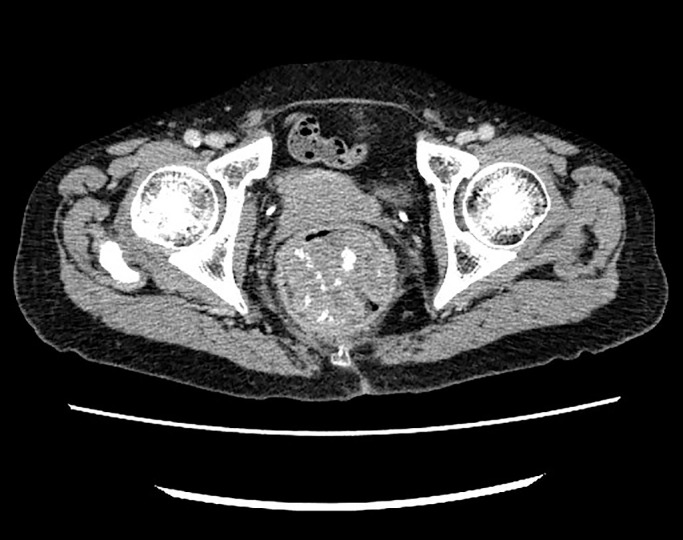
Abdominopelvic contrast-enhanced computed tomography shows irregular wall thickening on the pelvis with coarse heterotopic ossification.

Colonoscopy revealed stenosis with a protuberant mass 10 cm from the anal verge (Figure 1). Analysis of the biopsy specimen demonstrated adenocarcinoma without ossification. Abdominopelvic contrast-enhanced computed tomography (CT) showed irregular wall thickness of the rectum, multiple lymph node metastases, and liver metastases. CT revealed a rectal tumor with hyperintense calcified deposits (Figure 2). Subsequent emergency surgery was performed to treat intussusception and hemorrhage. We then performed Hartmann’s operation and D3 lymph node resection. Surgical time was 219 min and blood loss was approximately 100 g.

**Figure 3:**
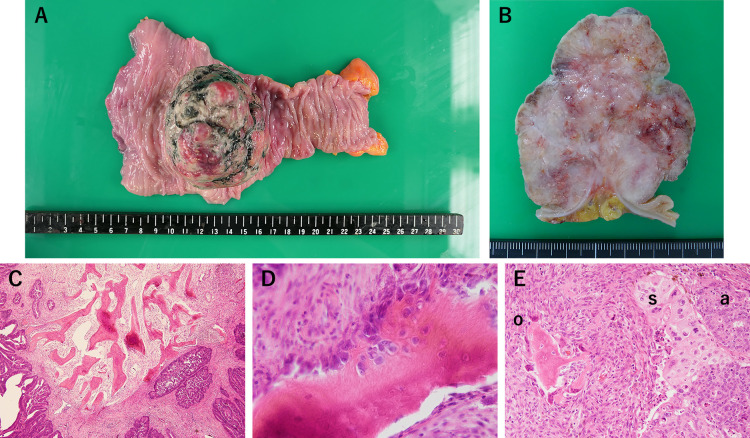
On macroscopic findings, (A) the surgical specimen contains a superficial protuberant mass. (B) The tumor extended into the subserosa through the muscular layer. On microscopic of H&E stain, (C) moderately differentiated adenocarcinoma with heterotopic ossification in the stroma. (D) Osteoblast cells were found around the HO. (E) This specimen was found HO(o), adenocarcinoma nest(a), and squamous cell carcinoma-like component(c). Osteoclast cells were found around the HO.

The surgical specimen contained a superficial protuberant mass (type 1), 9.0×7.0×6.0 cm. Microscopy revealed that the tumor extended into the subserosa through the muscle layer (Figures 3A, B). The tumor was diagnosed as a moderately to poorly differentiated adenocarcinoma with osseous metaplasia. The final pathological diagnosis was stage ⅣA adenocarcinoma (T3, N2a, M1a) in accordance with the 8th edition of the UICC TNM classification system. The specimen exhibited tubular and solid adenocarcinoma nests and squamous carcinoma-like components over a necrotic extent without secreted mucin. Benign ossified lesions observed in the stroma contained multiple trabecular bones, varying in size. Osteoblasts and osteoclasts were present around the HO (Figures 3C, D, E). HO was not detectable in four metastatic lymph nodes.

**Figure 4:**
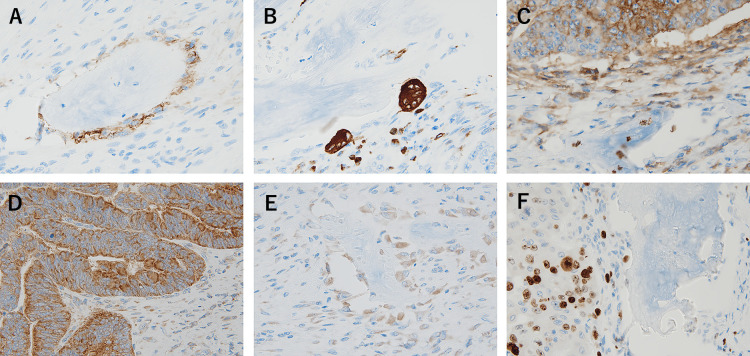
Findings of immunohistochemical stain. (A) Positive and diffuse membrane staining of osteoblast cells with CD56. (B) Strong positive cytoplasmic staining pattern of osteoclast cells with CD68. (C) The tumor cells and the stroma cells showed positive for CD44. (D) BMP-2 was observed to be strongly positive in tumor cells. (E) BMP-2 expression was weakly positive in the stromal cells around HO. (F) The tumor cells showed strongly positive for p53 in the nucleus, but the HO showed negative.

**Figure 5:**
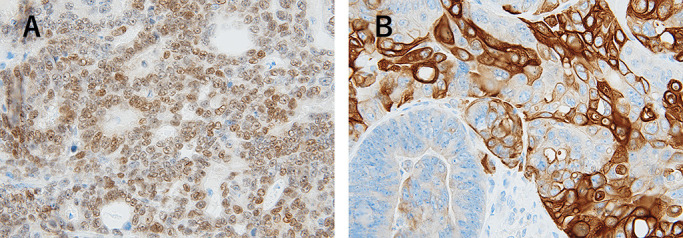
Findings of immunohistochemical stain. (A) Strong positive in primary rectal cancer cells staining with CDX2 in the nucleus. (B) Squamous carcinoma-like component showed strongly positive for 34βE12, but adenocarcinoma cells showed negative.

Immunohistochemical analysis of osteoid areas detected expression of CD56 in the membrane of osteoblasts and strong expression of CD68 in the cytoplasm of multinucleated osteoclasts. The tumor and the stromal cells expressed CD44 and vimentin. Bone morphogenetic protein 2 (BMP-2) was strongly expressed in tumor cells and weakly expressed in the stromal cells around the HO. Ki67 and p53 were expressed in tumor cells, but not in the osseous lesion (Figure 4). Strong nuclear expression of caudal-type homeobox 2 (CDX2) was detected in primary rectal cancer cells. Strong expression of a squamous cell carcinoma-like component was detected using the monoclonal antibody 34βE12 that did not detectably react with adenocarcinoma nests (Figure 5). However, p40 and p63 were undetectable in the carcinoma-like lesions (not shown). Biomarker analysis detected a mutation in codon 12 of *KRAS*, whereas *NRAS* and *BRAF* sequences were wild-type.

The patient was discharged on postoperative day 19. She received chemotherapy (mFOLFOX6 with bevacizumab) as the first option and was alive 5 months after surgery.

## Discussion

To the best of our knowledge, the present study is the first to identify HO in a primary rectal cancer with squamous cell carcinoma-like differentiation, which was surgically resected. HO is an unusual entity that is rarely encountered in rectal cancer. For example, only 19 reports of such cases are published, and 17 describe only primary tumors. One case had HO only in metastatic lymph nodes,^10^ while another had both. Liu^11^ et al. suggest an HO incidence of <0.15% in their hospital, with a male to female ratio=11:8, mean age 59.3 years. Furthermore, the most common primary site is the rectum, followed by the ascending or sigmoid colon, which is consistent with our case.

The mechanism of pathogenesis of HO is yet to be completely defined. For example, Dukes^8^ reported that HO is associated with long duration of symptoms, indicating slow growth of the tumor, growth of low-grade malignancy with little vascular invasion, and necrosis within the tumor. Accordingly, HO is formed by fibroblasts in the granulation tissue of necrotic nests; and calcium deposition adjacent to heterotopic ossification in mucin is suggested to play an important role in HO.^11–13^ Furthermore, osteoid matrices are observed in stromal and tumor cells, suggesting that factors secreted by tumor cells cause pluripotent stromal cells to metamorphose into osteoprogenitor cells.

The pathogenesis of HO associated with nonepithelial tissues may correlate with the bone-inducing properties of extracellular mucin and the activities of specific transcription factors, as well as the bone-evoking activity of the epithelium. Imai^14^ et al. suggest that transformation of stromal mesenchymal cells is induced by multifunctional BMPs, which are members of the transforming growth factor-β (TGF-β) superfamily. BMPs required for the formation of new bone include BMP-5 and BMP-6, which are expressed by tumor cells. In contrast, BMP-2 and -4 are strongly expressed in the surrounding mesenchymal cells, irrespective of weak expression in tumor cells. These findings suggest that tumor cells mainly produce BMP-5 and -6, which then induce the differentiation of surrounding mesenchymal cells into preosteoblasts and osteoblasts that express BMP-2 and -4. Therefore, HO found in the carcinoma is likely directly associated with the BMP produced via epithelial tumor cells.

Nagano et al. find that TGF-β1 in tumor cells stimulates the expression of nuclear Gli2 and that Gli2 stimulates the expression of BMP-2.^10^ BMP-2 induces bone formation and bone regeneration and induces the production of bone materials such as osteocalcin and osteonectin. Furthermore, tumor cells acquire the ability to express and secrete BMP-2 and TGF-β1, contributing to the transformation of fibroblasts and pluripotent stem cells into bone cells. Here we consistently observed osteoblasts around HOs, as well as osteoclasts, in some regions of the osseous lesion. However, we did not detect mucin deposits in tumor nests and the stroma.

Ossification involves the activation of osteogenic activities as well as the suppression of osteoclast function. CD44, which is strongly expressed in stromal cells and weakly expressed in tumor cells, inhibits osteoclast genesis depending on the microenvironment. Our case provided a suitable environment for osteogenesis. Furthermore, BMP-2 was strongly expressed by tumor cells and weakly expressed by stromal cells, suggesting that tumor cells strongly secrete BMP-2, which may have transformed fibroblasts and pluripotent stem cells into osteocytes. Furthermore, HO occurred only in primary tumor nests but not in lymph node metastases, suggesting that the development of HO requires optimal conditions for tumor cells to secrete osteogenic factors.

The histological types of at least 90% of cases of primary colorectal cancers comprise adenocarcinoma, mucinous adenocarcinoma, and micropapillary carcinoma. In striking contrast, 0.09%–0.5% of cases comprise adenosquamous carcinoma (ASC)/squamous carcinoma.^15^ Adenocarcinoma is the histological type of documented primary colorectal cancers with HO. However, in the present case, the squamous cell carcinoma-like areas strongly reacted with the anti-cytokeratin antibody 34βE12. In contrast, p40 and p60 were undetectable. Accordingly, we propose that the mechanism of histogenesis of primary ASC of the colon is as follows: undifferentiated or basal cells transform into squamous carcinoma cells, and cells of the ectopic aberrant squamous epithelium undergo malignant transformation. Consequently, ASC arises from metaplastic squamous cells in the mucosa or from adenocarcinoma cells. In the present case, adenocarcinoma developed into a squamous cell carcinoma-like lesion, considered squamous metaplasia of adenocarcinoma.

The prognosis of ASC of the colon is worse compared with that of adenocarcinoma (5-year overall survival rates of 23.5% vs 53.6%, respectively). Thus, the majority of ASC cases are detected at an advanced stage,^16^ and an aggressive tumor phenotype is typically associated with resistance to chemotherapy.^14^ In the present case, intussusception and hemorrhage of a rectal tumor required surgery without chemotherapy. Our patient underwent five cycles of mFOLFOX6 with bevacizumab therapy for 3 months after rectal resection. New metastases were undetectable, and the therapeutic outcome was judged as stable disease in accordance with the Response Evaluation Criteria Solid Tumors Guidelines (ver. 1.1). Moreover, previous studies show that ASC does not respond to 5-FU-based chemotherapy, accounting for the use of combined radiotherapy and chemotherapy (e.g., capecitabine with oxaliplatin in subsequent studies).^17,18^ We therefore plan to perform hepatectomy of liver metastases.

However, HO is classified as a non-neoplastic lesion associated with benign and malignant tumors. In our present case, HO occurred in a primary rectal carcinoma that did not detectably express p53 or Ki67, and these cells did not show atypia. Therefore, the presence of HO did not affect our decision to implement a treatment strategy appropriate for tumor stage. However, we reasoned that it is important to distinguish the bony lesion from osteosarcoma and chondrosarcoma. We continue to closely monitor this patient.
